# PulPy: A Python Toolkit for MRI RF and Gradient Pulse Design

**DOI:** 10.21105/joss.06586

**Published:** 2024-11-01

**Authors:** Jonathan B. Martin, Heng Sun, Madison Albert, Kevin M. Johnson, William A. Grissom

**Affiliations:** 1 Vanderbilt University Institute of Imaging Science, Vanderbilt University Medical Center, Nashville, United States of America; 2 Department of Biomedical Engineering, Yale University, New Haven, United States of America; 3 Department of Biomedical Engineering, Case Western Reserve University, Cleveland, United States of America; 4 Department of Medical Physics and Radiology, University of Wisconsin School of Medicine and Public Health, Madison, United States of America

## Summary

We present PulPy (Pulses in Python), an extensive set of open-source, Python-based tools for magnetic resonance imaging (MRI) radiofrequency (RF) and gradient pulse design. PulPy is a Python package containing implementations of a wide range of commonly used RF and gradient pulse design tools. Our implemented functions for RF pulse design include advanced Shinnar-LeRoux (SLR), multiband, adiabatic, optimal control, $B_{1}^{+}$-selective and small-tip parallel transmission (pTx) designers. Gradient waveform design functionality is included, providing the ability to design and optimize readout or excitation k-space trajectories (John Pauly et al., 1989). Other useful tools such as vendor-specific waveform input/output, Bloch equation simulators, abstracted linear operators, and pulse reshaping functions are included. This toolbox builds on the RF tools introduced previously in the SigPy.RF Python software package (Martin et al., 2020). The current toolbox continues to leverage SigPy’s existing capabilities for GPU computation, iterative optimization, and powerful abstractions for linear operators and applications (Ong & Lustig, 2019). The table below shows an outline of the implemented functions.

**Figure 1:**
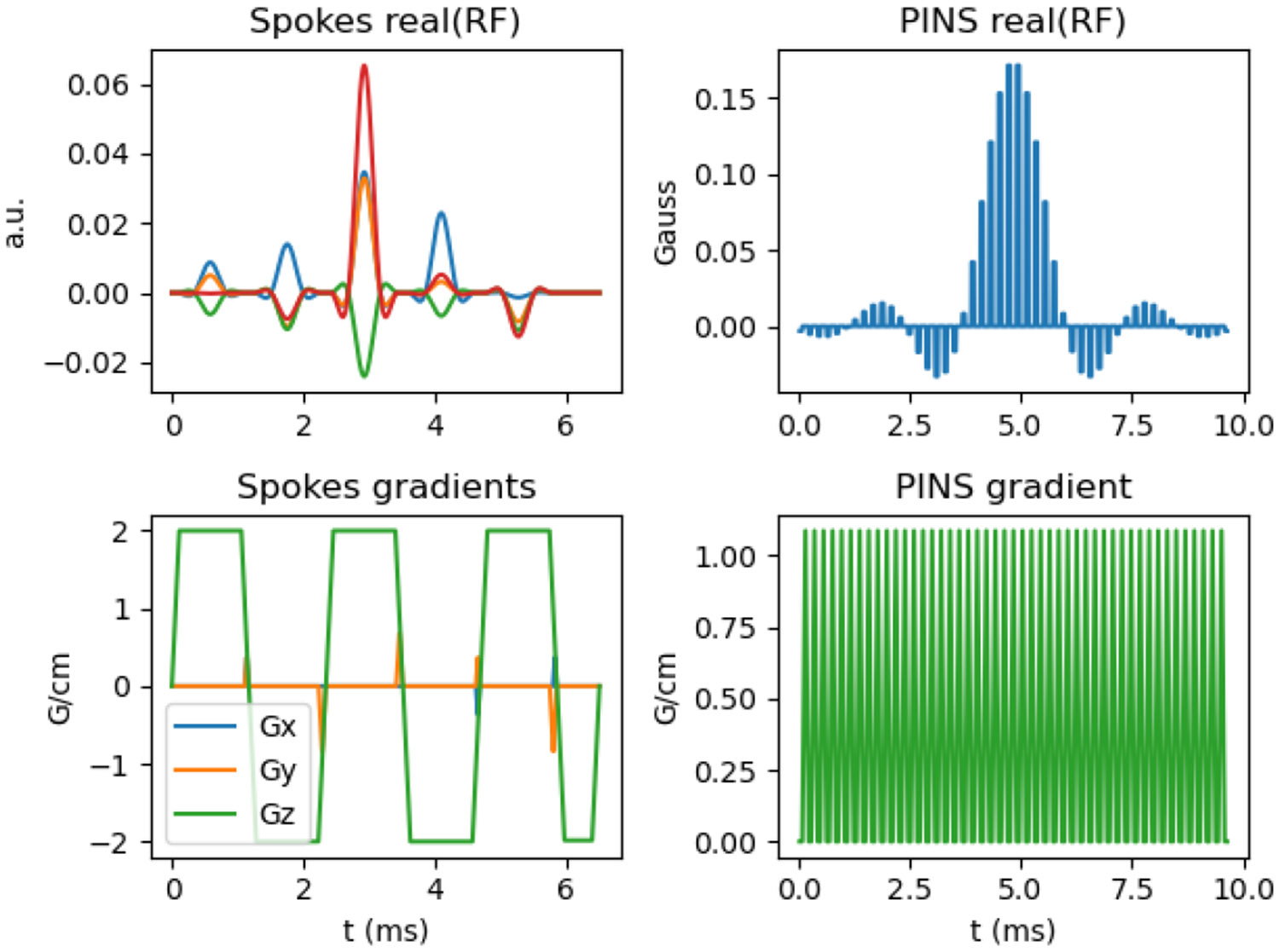
Example RF and gradient waveforms that PulPy can produce. Top left: 4-channel spokes RF pulse. Bottom left: associated 3-axis spokes gradient waveforms. Top right: PINS excitation RF pulse. Bottom right: associated slice-axis gradient

Preliminary development of this toolbox was presented in Martin et al. (2020). The pulse design tools were initially implemented as a sub-package in the SigPy Python package for signal processing and image reconstruction (Ong & Lustig, 2019). PulPy migrates those tools into a pulse design specific package, with SigPy as an external dependency. PulPy has been streamlined and expanded to include a larger collection of RF and gradient pulse design methods from the literature, as well as additional utility tools for I/O, pulse reshaping, and experimental $B_{1}^{+}$-selective pulse design algorithms. The toolbox has proved useful for prototyping novel pulse design algorithms, enabling the publication of Martin et al. (2022) by the authors and several works from other groups Wu et al. (2023). Figure 1 shows an example of RF and gradient waveforms produced by PulPy.

## Statement of need

The field of magnetic resonance imaging is currently experiencing rapid growth in available open source imaging tools. Tools have been made freely available for MRI hardware development (Amrein et al., 2022; Anand, 2018), system simulation (Stöcker et al., 2010; Villena et al., 2014), pulse sequence programming (Layton et al., 2017), image reconstruction (Ong & Lustig, 2019; Uecker et al., 2015), and post-processing and analysis (Avants et al., 2014; Duval et al., 2018; Soher et al., 2023). The great increase in open-source tools has helped enable fully open-source imaging systems (Arndt et al., 2017; Artiges et al., 2024). However, one critical aspect of the imaging pipeline which has seen limited open-source tool development is RF and gradient pulse design. While RF pulse and gradient designers increasingly share code online in independent repositories, there are few sets of common pulse design tools maintained in a rigorous and consistent manner with easy-to-read code and tutorials. This is despite the reality that in many cases, carefully designed or application-specific RF and gradient pulses are crucial to the success of MRI or NMR techniques. An open source pulse design code library would facilitate the development and dissemination of novel techniques and the comparison of approaches, similar to how BART (Uecker et al., 2015) and SigPy (Ong & Lustig, 2019) have made advanced parallel imaging and reconstruction methods widely accessible. To meet this need, we have developed a library of MRI pulse design tools. We call this new package PulPy, short for Pulses in Python.

## Target Audience

The PulPy toolbox has been developed for use by MRI researchers who are interested in pulse sequence design, MRI physics, signal processing, and optimization. We believe that it will serve as an essential building block for more general image acquisition tools which require specialized RF pulses. The previous iteration of this toolbox, SigPy.RF, has already been incorporated into open-source sequence development software such as Pulseq (Layton et al., 2017) and PyPulseq (Sravan Ravi et al., 2019) to provide RF pulses critical to the performance of various pulse sequences. We feel that PulPy, with its’ more specific focus on pulse design, will be able to even more easily integrated into other MRI acquisition software toolboxes, and we encourage other MRI software developers to incorporate PulPy as a component of MRI acquisition software. Finally, end-to-end optimization of MRI pulse sequences and reconstructions is being increasingly explored (Radhakrishna & Ciuciu, 2023; Wang et al., 2022); with the RF pulse and gradient waveform design functions provided, the PulPy package could facilitate this research.

Reproducibility and standardization are critical needs in MRI , and any method of reducing methodological variability is desirable. We believe that having centralized references for RF and gradient pulses will help promote consistency between studies by providing common code sources for the most widely used RF and gradient pulses. PulPy’s predecessor toolbox, SigPy.RF, also served as a hands-on teaching aid for researchers and students. An example is the educational ISMRM tutorial associated with SigPy.RF (Martin et al., 2020). This is a role that the PulPy toolbox will continue to fill. We have developed several tutorials, which are accessible to a wide audience with minimal prior MRI knowledge.

## Availability and Use

The latest version of PulPy includes the latest stable release of the pulse design tools and is available from the main repository. It can be installed through pip- see the documentation for more details. Jupyter notebook based pulse design tutorials for PulPy are also available, which demonstrate the toolbox being used for several classes of pulse design.

## Figures and Tables

**Table 1:** List of modules within PulPy and their basic functionality. Exemplary references are included.

Module	Description
.rf.adiabatic.py	Adiabatic/frequency-swept RF pulses e.g., Garwood (2001)
.rf.b1sel.py	B1-selective pulses e.g., Martin et al. (2022)
.rf.multiband.py	Pulses for simultaneous multi-slice e.g., Norris et al. (2011)
.rf.optcont.py	Large tip angle optimal control design e.g., Connolly et al. (1986)
.rf.ptx.py	parallel transmit pulse designers e.g., Grissom et al. (2006)
.rf.shim.py	parallel transmit RF shimming e.g., Mao et al. (2006)
.rf.slr.py	Conventional SLR and variations e.g., J. Pauly et al. (1991)
.rf.util.py	RF pulse design utilities
.grad.waveform.py	Gradient and trajectory designers e.g., Kim et al. (2003)
.grad.optim.py	Gradient and trajectory optimization e.g., Lustig et al. (2008)
io.py	Vendor-specific scanner input/output
linop.py	Linear operators for pulse design e.g., Grissom et al. (2006)
sim.py	1-D/N-D/N-coil Bloch simulation e.g., Mansfield & Morris (1982)
verse.py	RF pulse/gradient reshaping tools
